# Diabetes: Risk factor and translational therapeutic implications for Alzheimer's disease

**DOI:** 10.1111/ejn.15619

**Published:** 2022-02-23

**Authors:** Jeffrey Cummings, Andrew Ortiz, Janelle Castellino, Jefferson Kinney

**Affiliations:** ^1^ Chambers‐Grundy Center for Transformative Neuroscience, Department of Brain Health, School of Integrated Health Sciences University of Nevada Las Vegas (UNLV) Las Vegas Nevada USA; ^2^ Department of Brain Health, School of Integrated Health Sciences University of Nevada Las Vegas (UNLV) Las Vegas Nevada USA; ^3^ School of Medicine University of Nevada Las Vegas Las Vegas Nevada USA

**Keywords:** Alzheimer's disease, dapagliflozin, diabetes, empagliflozin, GLP‐1 agonist, insulin, liraglutide, metformin, mouse model, pioglitazone, rosiglitazone, semaglutide

## Abstract

Type 2 diabetes mellitus (T2DM) and Alzheimer's disease (AD) commonly co‐occur. T2DM increases the risk for AD by approximately twofold. Animal models provide one means of interrogating the relationship of T2DM to AD and investigating brain insulin resistance in the pathophysiology of AD. Animal models show that persistent hyperglycaemia results in chronic low‐grade inflammation that may contribute to the development of neuroinflammation and accelerate the pathobiology of AD. Epidemiological studies suggest that patients with T2DM who received treatment with specific anti‐diabetic agents have a decreased risk for the occurrence of AD and all‐cause dementia. Agents such as metformin ameliorate T2DM and may have other important systemic effects that lower the risk of AD. Glucagon‐like peptide 1 (GLP‐1) agonists have been associated with a decreased risk for AD in patients with T2DM. Both insulin and non‐insulin anti‐diabetic treatments have been evaluated for the treatment of AD in clinical trials. In most cases, patients included in the trials have clinical features of AD but do not have T2DM. Many of the trials were conducted prior to the use of diagnostic biomarkers for AD. Trials have had a wide range of durations and population sizes. Many of the agents used to treat T2DM do not cross the blood brain barrier, and the effects are posited to occur via lowering of peripheral hyperglycaemia and reduction of peripheral and central inflammation. Clinical trials of anti‐diabetic agents to treat AD are ongoing and will provide insight into the therapeutic utility of these agents.

## Graphical abstract

T2DM and AD are linked in several important ways. T2DM is a risk factor for AD. T2DM is characterized by chronic low‐grade inflammation, and there is evidence linking peripheral inflammation to central inflammation. Hyperglycaemia in animal models produces AD‐related changes. Neurons in AD have insulin resistance. Agents that lower blood sugar and peripheral inflammation may have beneficial effects on AD. Clinical trials of insulin and other anti‐diabetic agents have not shown benefit in patients with AD; additional trials are on‐going.

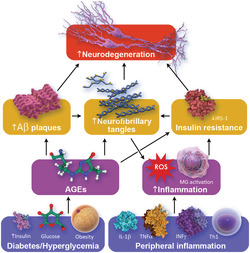

**Abbreviations and acronyms**
AD: Alzheimer's disease
ADAS‐cog: Alzheimer's Disease Assessment Scale – cognitive subscale
ADAS‐Exec: AD Assessment Scale – Executive version
ADCS‐ADL: Alzheimer's Disease Cooperative Study–Activities of Daily Living Scale
AGE: advanced glycation end‐products
AKT: protein kinase B
AMP: adenosine monophosphate
AMPA: α‐amino‐3‐hydroxy‐5‐methyl‐4‐isoxazolepropionic acid
AMPK: AMP‐activated protein kinase
APOE: apolipoprotein E
APP: amyloid precursor protein
Aβ: amyloid beta
BACE: β‐site APP‐cleaving enzyme
BBB: blood–brain barrier
BDNF: brain‐derived neurotrophic factor
CaMKII: calcium/calmodulin‐dependent protein kinase II
cdk5: cyclin‐dependent kinase 5
CDR‐SB: Clinical Dementia Rating ‐ Sum of Boxes
CIBIC: Clinician's Interview‐Based Impression of Change with Caregiver Input
CNS: central nervous system
CRP: C‐reactive protein
CSF: cerebrospinal fluid
DM: diabetes mellitus
DNA: deoxyribonucleic acid
EGFP: enhanced green fluorescence protein
ELAD: evaluating liraglutide in Alzheimer's disease
EV: extracellular vesicles
EVOKE: A Research Study Investigating Semaglutide in People with Early Alzheimer's Disease
FABP: fatty acid‐binding protein
fAD: familial Alzheimer's disease
FDG: fluorodeoxyglucose
GABA: γ‐aminobutyric acid
GIP: glucose‐dependent insulinotropic polypeptide
GLP: glucagon‐like peptide
GSK‐3β: glycogen synthase kinase‐3β
GWAS: genome‐wide association studies
HDL: high‐density lipoprotein
HFD: high‐fat diet
HgbA1c: haemoglobin‐A1c
HLA: human leukocyte antigen
ICV: intracerebroventricular
IDE: insulin‐degrading enzyme
IFG: insulin‐like growth factor
IL: interleukin
INF‐gamma: interferon gamma
IR: insulin receptor
IRS: insulin receptor substrate
JNK: c‐Jun N‐terminal kinase
LOAD: late onset Alzheimer's disease
LPS: lipopolysaccharide
LTD: long‐term depression
LTP: long‐term potentiation
MAPK: mitogen‐activated protein kinase
MCI: mild cognitive impairment
MMSE: Mini‐Mental State examination
MRI: magnetic resonance imaging
MWM: Morris water maze
NFT: neurofibrillary tangles
NGS: next‐generation sequencing
NIA: National Institute on Aging
NMDA: *N*‐methyl‐*d*‐aspartate
NO: nitric oxide
PET: positron emission tomography
PGE2: prostaglandin E2
PI3K: phosphatidylinositol 3‐kinase
PI3K: phosphoinositide 3 kinase
PI3K/AKT: phosphatidylinositol 3‐kinase/protein kinase B
PKA: protein kinase A
PPARγ: peroxisome proliferator‐activated receptor γ
PPY: pancreatic polypeptide
PS1: presenilin 1
PS2: presenilin 2
RAGE: receptor advanced glycation end‐products
ROS: reactive oxygen species
SRT: selective reminding test
STZ: streptozotocin
T1DM: type 1 diabetes mellitus
T2DM: type 2 diabetes mellitus
Th1: T helper cells
TLR: toll‐like receptor
TNF: tumour necrosis factor
TZD: thiazolidinediones
WT: wild type
XR: extended release

## 1 INTRODUCTION

Alzheimer's disease (AD) is a neurodegenerative disease that is characterized by progressive synaptic and neuronal loss, learning and memory deficits, and cognitive, functional, and behavioural decline (Scheltens et al., 2021). AD is the most common form of dementia, accounting for 60%–80% of all cases (Alzheimer's Association, 2021). Approximately 6.2 million Americans and nearly 50 million individuals worldwide have AD dementia (Alzheimer's Association, 2021); AD is the sixth leading cause of death in the United States (Alzheimer's Association, 2021). Age is the greatest risk factor for developing AD, although AD is not a normal part of aging (Alzheimer's Association, 2021). Several other factors confer increased risk including apolipoprotein E ε‐4 (APOE4) genotype, stroke and vascular risk factors, head trauma, diabetes mellitus (DM), and obesity (Barnes & Yaffe, 2011). Type 2 diabetes mellitus (T2DM) can confer a 1.5‐fold to fourfold increase in lifetime risk for AD (Lu et al., 2009; Mehla et al., 2014). Furthermore, approximately 80% of individuals with AD have insulin resistance or abnormal fasting glucose levels (Janson et al., 2004). Over 25% of the US population over the age of 65 have T2DM, and aging of the population plays a large role in both the epidemic of T2DM and the increased prevalence of AD (Kirkman et al., 2012). Understanding the mechanistic relationships of T2DM and AD may lead to candidate treatments that control T2DM and ameliorate the risk of progression to AD. Some of these agents may treat AD independent of the occurrence of T2DM.

The pathological hallmarks of AD include amyloid‐beta (Aβ) protein plaques composed of fibrillar Aβ (Hardy & Higgins, 1992; Hyman et al., 2012), neurofibrillary tangles (NFTs) composed of hyperphosphorylated tau protein (Grundke‐Iqbal et al., 1986; Serrano‐Pozo et al., 2011), and chronic neuroinflammation, or a sustained immune response in the brain, that promotes and accelerates both Aβ and tau pathologies (Heneka et al., 2015; Kinney et al., 2018; Millington et al., 2014).

Here, we review the foundational science linking T2DM and AD, animal models used to explore the relationship of the two disorders, and past and current clinical trials of diabetes therapies tested for the treatment of AD.

### 1.1 Foundational science links between T2DM and AD

Many types of evidence link T2DM and AD. T2DM affects over 200 million people worldwide and is defined as a sustained state of hyperglycaemia due to dysfunction of insulin receptor (IR) signalling (insulin resistance) despite elevated levels of insulin (Ahmed, 2012). Several studies have shown that insulin resistance is a risk factor for AD and may aggravate the pathology of AD (Boles et al., 2017; de Felice, 2013; Ferreira et al., 2018; Talbot et al., 2012a). Altered insulin signalling disrupts brain function, as insulin in the brain promotes neurite growth, synaptic plasticity, and development and maintenance of excitatory synapses (Gu et al., 2014; Liu et al., 2014; Taouis & Torres‐Aleman, 2019; Zhao, Siu, et al., 2019). Insulin administration via oral, nasal or intracerebroventricular (ICV) injections decreases AD pathology in animal models of T2DM with AD‐like changes (Adzovic et al., 2015; Avgerinos et al., 2018; Cummings et al., 2020; Freiherr et al., 2013; Morris & Burns, 2012; Steinmetz et al., 2016).

Metabolic syndrome is composed of insulin resistance, obesity, hypertension, cardiovascular disease and dyslipidaemia which increase the risk of AD beyond the risk conferred by T2DM (Hildreth et al., 2012; Verdile et al., 2015). Obesity, T2DM and AD have overlapping biological pathologies including insulin resistance, oxidative stress, mitochondrial dysfunction and inflammation (Pugazhenthi et al., 2017).

Insulin interacts with tau pathology via the activation of protein kinases involved in tau phosphorylation. Activation of kinases including protein kinase A (PKA), calcium/calmodulin‐dependent protein kinase II (CaMKII), glycogen synthase kinase‐3β (GSK‐3β) and cyclin‐dependent kinase 5 (cdk5) leads to the hyperphosphorylation of tau and aggregation of tau proteins comprising NFTs (Dolan & Johnson, 2010; Duka et al., 2013; Engin & Engin, 2021; Wang et al., 2007). GSK‐3β is a serine‐threonine kinase that is consistently upregulated in AD brains (Blalock et al., 2004; Hooper et al., 2008; Leclerc et al., 2001; Lovestone et al., 1994; Munoz‐Montano et al., 1997). GSK‐3β can contribute to development of NFTs (Beurel et al., 2015; Hanger et al., 1992; Mandelkow et al., 1992) and Aβ plaque formation (Beurel et al., 2015; Hurtado et al., 2012; Phiel et al., 2003). In a reciprocal relationship, Aβ aggregation appears to promote tau hyperphosphorylation via activation of GSK‐3β (Reddy, 2013).

Animal models are used to explore the mechanistic relationships of T2DM and AD. Streptozotocin (STZ), for example, when given in staggered and low‐dose injections, destroys pancreatic β cells mimicking late‐stage T2DM pancreatic exhaustion (McEvoy et al., 1984; Murtishaw et al., 2018; Reed et al., 2000; Srinivasan et al., 2005; Zhang et al., 2008). Treated animals have sustained hyperglycaemia without any overt illness (Murtishaw et al., 2018); they exhibit learning and memory deficits, increased tau phosphorylation and increased neuroinflammation similar to the pathological changes observed in AD (Murtishaw et al., 2018).

Signalling pathways link T2DM to AD. Fractalkine (CX3CL1) is a chemokine constitutively and specifically released by neurons to regulate microglia involved in neuroinflammation through the fractalkine receptor (CX3CR1) located on central nervous system (CNS) microglia (Chamera et al., 2020). CX3CL1/CX3CR1 signalling interacts with insulin regulation in the brain by modulating microglial function and hippocampal synaptic plasticity (Paolicelli et al., 2014; Sheridan et al., 2014). A tau transgenic (Tg) mouse model (hTau mice) lacking CX3CR1 exhibited enhanced tau phosphorylation and aggregation associated with microglial activation, as well as behavioural impairments (Bhaskar et al., 2010; Bolos et al., 2017).

Genetic factors suggest a link between T2DM and AD. APOE‐4 is a gene encoding a protein that is involved in lipid and cholesterol binding and transport and is the best‐known genetic risk factor for AD (Corder et al., 1993; Kim et al., 2009; Strittmatter et al., 1993). Two copies of the ε4 allele of the gene can confer up to a 15‐fold increase in developing AD (Altmann et al., 2014; Farrer et al., 1997; Payami et al., 1994). APOE‐4 has been shown to increase hyperinsulinaemia in T2DM amplifying the risk for AD in APOE‐4 carriers with T2DM (Luchsinger et al., 2004; Peila et al., 2002).

### 1.2 Insights from animal models of AD and T2DM

Most of the work on AD‐related mechanisms as well as the testing of candidate therapeutics prior to clinical trials is carried out in animal models that mimic aspects of AD; this has largely relied on the use of genetic mouse models that express mutations associated with familial AD (fAD). These models present some of the core pathological features of AD but do not exhibit the entire range of AD pathology.

Animal models remain indispensable in research due to their biological similarity to humans, allowing researchers to investigate disease mechanisms and progression under controlled conditions. They continue to advance both the scientific and medical fields and provide the scientific basis for the creation of novel therapeutics. Approximately 60% of all preclinical research is conducted in rodents due to their anatomical, physiological, genetic and overall biological similarity to humans (Bryda, 2013). They allow researchers to target and alter specific genes, translational pathways and protein interactions that provide insights into mechanisms and facilitate testing potential therapeutics. Several model‐based approaches have been utilized to investigate mechanisms and features of AD and T2DM both independently and together.

### 1.3 Common AD mouse models/mechanisms and use in metabolic and diabetes investigations

There is no animal model that exactly mimics AD pathogenesis and progression. Wild‐type (WT) mice do not normally develop AD‐type pathology; hence, human genes consistent with fAD (e.g., amyloid precursor protein [APP], presenilin 1 [PS1] and presenilin 2 [PS2]) are commonly incorporated into mouse genomes to induce AD pathology in Tg mouse models (Balu et al., 2019). The AD pathology of fAD and sporadic late onset AD (LOAD) are morphologically similar. It is common in a laboratory setting to utilize several fAD genetic mutations to create AD animal models. There are currently over 200 animal models of AD tracked by Alzforum (https://www.alzforum.org/research-models/alzheimers-disease), which include models facilitating the pathogenesis of Aβ plaques, NFTs, gliosis, synaptic loss, neuronal loss, changes in long‐term potentiation and long‐term depression (i.e., LTP/LTD, respectively), and cognitive impairment. The APP/PS1 mouse, for example, is arguably the most widely used animal model to investigate Aβ pathology progression and other features of AD. The APP/PS1 model contains mutations in APP (Swedish) and PSEN1 (L166P) genes, under the Thy1 promotor, and express a threefold increase in APP production compared with endogenous murine APP. APP is the precursor protein for Aβ, and these mice develop Aβ plaques in the cortex and hippocampus at approximately 3–4 months of age (Radde et al., 2006), followed by reactive gliosis and proinflammatory cytokine release (Lee et al., 2010; Radde et al., 2006), synaptic loss (Bittner et al., 2012), neuronal loss in the dentate gyrus (Rupp et al., 2011), hippocampal LTP/LTD impairments (Gengler et al., 2010) and cognitive impairment (Webster et al., 2013), all consistent with AD. APP/PS1 mice are often utilized for AD‐related studies because they closely mimic the progression of Aβ in AD patients.

Studies of the relationship between AD and T2DM often rely on manipulations that alter diet, blood glucose, and overall metabolic functions produced artificially in animal models of AD.

### 1.4 Insulin resistance and AD

It has traditionally been proposed that reduction of brain metabolism occurs after neuronal atrophy and loss in the course of AD (Bokde et al., 2001). However, accumulating evidence indicates that hypometabolism, potentially because of metabolic dysfunction (e.g., insulin resistance observed in T2DM), may occur prior to brain atrophy (Kyrtata et al., 2021). Animal models are utilized to investigate the mechanisms by which this may occur. In mouse models, long‐term feeding with a high‐fat diet (HFD) can cause hyperinsulinaemia, cardiovascular disease, obesity and both peripheral and central insulin resistance (Buettner et al., 2007; Wali et al., 2020). The HFD and obesity have been shown to increase neuroinflammation (Spagnuolo et al., 2015), neurodegeneration (Mazon et al., 2017; Pugazhenthi et al., 2017) and cognitive decline (Balasubramanian et al., 2021; Nguyen et al., 2014) that are consistent with AD. In clinical populations, most, but not all, patients with T2DM are overweight or obese, leading to insulin resistance. Insulin resistance and hyperglycaemia can induce hyperphosphorylated tau protein accumulation and lead to NFTs (Silva et al., 2019). The exact effects of diet‐induced insulin resistance on AD pathology and cognition remain controversial due to differing diet compositions, incubation times, cohort sex and rodent strains used in the experiments. The strength of these approaches is that they reproduce the milieu of changes observed with T2DM; however, they provide limited insight in to specific mechanisms linking T2DM and AD.

Genetic approaches to producing insulin resistance include the *ob/ob* mouse that is widely utilized to induce obesity and facilitating examination of diet‐induced insulin resistance. This rodent model, in a C57BL/6J background, is unable to produce leptin—a hormone responsible for inhibiting hunger—via altering the leptin gene (i.e., *ob* or *Lep*), leading to increased appetite and obesity (Ingalls et al., 1950; Small et al., 2017). These animals exhibit hyperinsulinaemia, mild hyperglycaemia and insulin resistance (Coleman, 1978; Small et al., 2017). Crossing the *ob/ob* mouse model with APP/PS1 mice (i.e., APP/PS1 + *ob/ob*) leads to models that exhibit significant increases in Aβ plaque load in the hippocampus and prefrontal cortex; two brain regions affected early in AD (S. Zhang et al., 2017). APP/PS1 + *ob/ob* mice evidence more abundant hyperphosphorylated tau, neuroinflammation (i.e., activated astrocytes and microglia) and synaptic loss and have more severe learning and memory deficits compared to APP/PS1 mice (Zhang et al., 2017).

Over the last 15 years, considerable attention has been devoted to understanding the sustained inflammatory response seen in the brain in AD. Chronic inflammation and insulin resistance are observed in both T2DM and AD (O'Brien et al., 2017; Parimisetty et al., 2016; Vinuesa et al., 2021). Systemic inflammation may precede the development of insulin resistance in insulin‐sensitive tissue (Klöting & Blüher, 2014; Parimisetty et al., 2016). In addition to peripheral and central inflammation in both T2DM and AD, there is evidence of inflammation‐related changes in the blood–brain barrier (BBB) in both conditions. The BBB is a neurovascular unit that limits peripheral toxins, immune cells and pathogens from entering and damaging the brain (van Dyken & Lacoste, 2018). Chronic peripheral and inflammation induced by DM and obesity can cause BBB breakdown and permeability to infiltrating macrophages, leading to exacerbation of the immune response in the brain, disruption of glial and neuronal cell integrity, impaired hormonal function, increased insulin insensitivity and impaired cognition (van Dyken & Lacoste, 2018). The invasion of leukocytes releases proinflammatory cytokines that exacerbate AD pathology including neuronal damage and death (Kinney et al., 2018; Varatharaj & Galea, 2017). In animal models of AD, chronic neuroinflammation can be seen before Aβ accumulation in the hippocampus (Beauquis et al., 2013; Heneka et al., 2015). Several glial signalling cascades implicated in regulation of immune responses are altered in AD and T2DM. For example, Toll‐like receptor 4 (TLR4) and triggering receptor expressed on myeloid cells 2 (TREM2) are expressed on microglia that regulate inflammation in the brain (Zhou et al., 2019). Activation of TLR4 produces large increases of proinflammatory cytokines such as tumour necrosis factor‐α (TNF‐α), nitric oxide (NO), prostaglandin E2 (PGE2) and interleukin‐1β (IL‐1β), which can promote and exacerbate both T2DM and AD pathology (Murtishaw et al., 2016; Zhao, Bi, et al., 2019). Lipopolysaccharide (LPS), an immunostimulatory component of gram‐negative bacteria, and a ligand for TLR‐4, induces inflammation in the brain (Zhao, Bi, et al., 2019). LPS‐induced CNS inflammation in 3xTg‐AD mice showed significant increase of tau hyperphosphorylation via cdk5 (Kitazawa et al., 2005). LPS‐induced inflammation following ICV injection of STZ induces IR insensitivity in the brain, increased tau phosphorylation in the hippocampus and learning and memory deficits in male Sprague–Dawley rats assessed in the Morris mater maze (MWM) (Murtishaw et al., 2016). These studies suggest that chronic inflammation as a result for T2DM, may be one mechanism by which T2DM confers increased risk for developing LOAD.

Additional signalling cascades that regulate both neuronal and glial function are altered in T2DM and AD. c‐Jun N‐terminal kinases (JNKs) are members of the mitogen‐activated protein kinase (MAPK) family and are involved in cellular stress responses; their activity has been linked to T2DM. JNK is among the most investigated molecules in obesity‐induced insulin resistance (Pal et al., 2016). JNK has three genetic isoforms, MAPK8 coding for JNK1, MAPK9 coding for JNK2 and MAPK10 coding for JNK3 (Yarza et al., 2016). JNK3 has been implicated in the development of AD (Yarza et al., 2016). It is highly expressed and chronically active in brain tissue and cerebrospinal fluid (CSF) in AD patients and has been implicated in the cognitive deficits consistent with AD (Gourmaud et al., 2015). Several downstream cascades that arise from JNK signalling are linked to cell survival and pathologic features of AD. JNK can directly induce insulin resistance through a phosphorylated IR substrate (IRS) 1, inhibiting insulin cascades and potentially increasing the risk for AD (Sabio et al., 2008). ICV injection of Aβ oligomers can activate IRS‐1pSer and JNK in the hippocampus of cynomolgus monkeys (Bomfim et al., 2012). Administration of exendin‐4—an anti‐diabetic agent—to Tg mice, decreased IRS‐1pSer and activated JNK, resulting in amelioration of behavioural and cognitive deficits (Bomfim et al., 2012). Mice given HFD to induce obesity exhibit JNK activation. Genetic manipulations leading to depletion of JNK prevent obesity by decreasing adiposity and ameliorating insulin sensitivity and IR signalling (Hirosumi et al., 2002). Genetic‐based depletion of JNK3 in fAD mice leads to a dramatic reduction of Aβ42 peptide and Aβ plaque load and improved cognition (Yoon et al., 2012). Aβ42 can indirectly activate JNK signalling, resulting in neuroinflammation and neurodegeneration (Yoon et al., 2012). JNK modulates NFT formation via phosphorylating tau (Lagalwar et al., 2006; Yarza et al., 2016). JNK‐related signalling represents a specific conserved pathway that intersects with obesity, insulin resistance and cell survival in AD and T2DM.

Taken together, insulin resistance may not directly cause AD; however, insulin resistance can increase the risk of developing AD by exacerbating AD pathology (e.g., Aβ, tau and chronic neuroinflammation) and, in turn, promote further insulin resistance—a feed‐forward loop (Wei et al., 2021). Therapeutics targeting insulin resistance may be useful to ameliorate this mechanism and reduce the risk of AD and the exacerbation of AD in those with existing pathological changes.

### 1.5 Hyperglycaemia and AD

Hyperglycaemia is a major characteristic of T2DM and has a high prevalence in aged populations. Twenty‐five percent of the US population over the age of 65 have T2DM (as defined by hyperglycaemia), and another ~88 million in the United States exhibit pre‐DM hyperglycaemia (elevated blood glucose but not yet meeting T2DM criteria; Centers for Disease Control and Prevention, 2020). In clinical populations, disruption of glucose homeostasis expedites the progression from mild cognitive impairment (MCI) to AD (Morris et al., 2014), suggesting that dysregulation of glucose metabolism may play a causal role AD pathogenesis (Macauley et al., 2015).

Hyperglycaemia can be modelled in in rodents with STZ, a diabetogenic drug that is toxic to insulin‐producing pancreatic β cells via the alkylation of β‐cell DNA, thus impairing insulin secretion and inducing chronic hyperglycaemia (Murtishaw et al., 2018). This approach has been useful in studying T1DM; however, the severity of the beta cell loss does not mimic T2DM seen in aging populations. To better approximate T2DM, investigators have developed staggered and/or low‐dose injections of STZ to effectively induce a progressive long‐term hyperglycaemia state in an otherwise healthy animal (Murtishaw et al., 2018). Investigations have demonstrated that staggered administration of STZ results in a sustained hyperglycaemic state with induction of learning and memory deficits, increased tau phosphorylation and increased neuroinflammation in mice consistent with AD pathological changes (Murtishaw et al., 2018). Investigations employing central STZ administered via a single ICV injection revealed exacerbation of Aβ accumulation in APP/PS1 mice (Kelliny et al., 2021). The central STZ administration is hypothesized to induce IR resistance centrally, potentially mimicking an important aspect of AD. Peripheral STZ and the resulting hyperglycaemia have been shown to increase APP protein expression in APP/PS1 mice, thus promoting Aβ generation (Yang et al., 2013) and directly exacerbate Aβ and tau pathologies (Arnold et al., 2018; Ferreira et al., 2018; Murtishaw et al., 2018; Yang et al., 2013). Acute hyperglycaemia in APP/PS1 mice can increase Aβ production in the hippocampal interstitial fluid, and the effect is exacerbated with increased age (Macauley et al., 2015). Pdx1+/− mice (a chronic hyperglycaemia mouse model) crossed with an APP/PS1 mouse exhibited increased tau phosphorylation, increased synaptic loss in the hippocampus, increased microglial and astrocyte activation, glucose intolerance and Aβ plaque formation (Guo et al., 2016). These mice exhibited increased advanced glycation end‐products (AGEs) followed by the activation of its receptor (RAGE), which is thought to contribute to impairments in Aβ degradation and Aβ generation (Guo et al., 2016). AGEs can induce synaptic and neuronal death via increased APP processing (i.e., β‐site APP‐cleaving enzyme [BACE] and PS1) and reactive oxygen species (ROS) generation (Ko et al., 2015). Elevated glucose levels can facilitate the formation of Aβ‐42 oligomers (the more toxic form of Aβ) (Kedia et al., 2017). It is hypothesized that glucotoxicity via chronic hyperglycaemia can induce neuronal structural and functional alterations, haemorrhagic interruption of cerebral blood vessels and increased Aβ accumulation. Glucotoxicity can result in cell injury to hepatocytes and insulin‐producing pancreatic β cells via mitochondrial oxidative stress and mitochondrial dysfunction (Lee, Lee, et al., 2011; Mota et al., 2016). Oxidative stress can increase the activity of BACE and gamma secretase, enzymes directly involved in the cleavage of APP and the generation of Aβ (Cheignon et al., 2017; Y. Zhao & Zhao, 2013).

Chronic hyperglycaemia induces hyperphosphorylation of tau via several kinases (Murtishaw et al., 2018; Wang et al., 2007). Activation of PKA, CaMKII, GSK‐3β and cdk5 in T2DM leads to the hyperphosphorylation of tau, aggregation of tau proteins and formation of NFTs seen in AD (Dolan & Johnson, 2010; Duka et al., 2013; Engin & Engin, 2021; Wang et al., 2007). Aβ accumulation appears to promote tau hyperphosphorylation via activation of GSK‐3β (Reddy, 2013). Chronic hyperglycaemia can induce tau modification via tau cleavage, both in vitro and in vivo (Kim et al., 2013). Thus, kinase alterations seen in T2DM can contribute to AD pathogenesis. The investigations of hyperglycaemia provide mechanistic evidence for how T2DM confers increased risk for developing AD.

### 1.6 Genetic relationships of T2DM and AD

#### 1.6.1 Apolipoprotein E

APOE is a protein that is involved in lipid and cholesterol binding and transport and is the most common genetic risk factor for AD (Corder et al., 1993; Kim et al., 2009; Strittmatter et al., 1993). In humans, there are three APOE alleles (i.e., E2, E3 and E4) that produce apo‐E2, apo‐E3 and apo‐E4 proteins. Approximately ~60% of AD patients have an APOE4 genotype (Rebeck et al., 1993). Having two copies of the ε2 allele is the strongest genetic protective factor for LOAD, whereas two copies of the ε4 variant can confer up to a 15‐fold increase risk in developing LOAD (Altmann et al., 2014; Farrer et al., 1997; Payami et al., 1994; Riedel et al., 2016; Serrano‐Pozo et al., 2021). Mice express only one form of APOE, and the amino acid homology between mouse and human APOE is 70% (Rajavashisth et al., 1985). APOE reporter mice with enhanced green fluorescence protein (EGFP) insertion reveal that microglia and astrocytes constitutively express APOE, and neurons synthesize APOE under stress conditions (Xu et al., 2006). Despite the increased risk in APOE‐4 human carriers, Tg fAD mice do not closely mimic the effects of human APOE isoforms (Balu et al., 2019), and a majority of Tg fAD mouse models use murine APOE instead of human APOE; this presents a limitation when investigating Aβ accumulation, synaptic integrity and neuroinflammation seen in AD (Balu et al., 2019). Given the large role that APOE plays in AD pathophysiology, several animal models of both DM and AD with APOE modifications have been investigated.

Although the APOE 4 gene significantly increases the likelihood of developing AD, many individuals who are APOE4 carriers do not develop the disease; it is hypothesized that APOE4 interacts with several factors, including obesity, that increase AD risk (Moser & Pike, 2017). For example, APOE4 has been shown to increase hyperinsulinaemia and T2DM, contributing to the risk of AD (Luchsinger et al., 2004; Peila et al., 2002). Diet‐induced obesity (i.e., western diet) in 5xfAD/human APOE‐ε4^+/+^ mice exhibit a significant increase in amyloid deposits, Aβ burden, and reactive gliosis compared to 5xfAD/human APOE‐E3^+/+^; suggesting that there is an interaction between obesity and APOE in increasing AD pathogenesis (Moser & Pike, 2017). Mice with human APOE4 with HFD‐induced insulin resistance replicate diabetic‐related states such as increased glucose and insulin resistance and decreased insulin secretion (Koren‐Iton et al., 2020). When mice with human APOE3 are fed a HFD, they show similar results as APOE4 mice, suggesting that diabetic modifications play an important role in the pathological effects of APOE (Koren‐Iton et al., 2020).

#### 1.6.2 Non‐APOE risk genes for AD and DM

Genome‐wide association studies (GWAS), next‐generation sequencing (NGS) and other technological advances point to several additional genetic loci, rare genetic variants and mutations that have a role in LOAD (Giri et al., 2016). These techniques have helped identify genetic influences that vary from low risk (e.g., CR1, CD33, CD2AP, etc.), to medium risk (e.g., ADAM12, PLD3, ABCA7, etc.), high risk (e.g., APOE, TREM2 and SORL1, etc.) and causal (e.g., APP, PS1 and PS2) associations with AD. Over 280 autosomal dominant variants have been observed in AD (Aguilar et al., 2019; Cruts et al., 2012; Yamazaki et al., 2016). The impact of genes influencing JNK and GSK‐3β discussed above suggest overlapping changes relevant to T2DM and AD.

Similarly, there are numerous candidate genes implicated in DM that may have direct linkage to AD pathogenesis. Risk genes that are strongly associated with T1DM include human leukocyte antigen (HLA) HLA‐DR3‐DQ2 or HLA‐DR4‐DQ8 haplotypes (Pociot & Lernmark, 2016). Over 50 genes, 58 genomic regions and more than 100 single nucleotide polymorphisms (SNPs) are associated with T1DM (Paschou et al., 2018; Pociot & Lernmark, 2016). Individuals with T1DM, especially the elderly, have an increased risk of developing AD (Lacy et al., 2018).

Genes strongly associated with T2DM include TCF7L2 (involved in insulin secretion and glucose production), ABBC8 (regulates insulin), CAPN10 (involved in insulin sensitivity and secretion), GLUT2 (transports glucose into pancreatic β cells), GCGR (involved in glucagon regulation) (Naseri et al., 2020) and others (Naseri et al., 2020; Park, 2011). These genes are involved in the overall production and regulation of glucose and insulin and in how glucose is sensed in the body; dysfunction of these processes can lead to T2DM and in parallel, increased risk for AD.

Some of these genes are known to link T2DM and AD. For example, the TCF7L2 gene is strongly associated with T2DM (Grant et al., 2006), and increased TCF7L2 mRNA has been observed in AD brains (Blom et al., 2010). ABCA1 regulates cholesterol efflux and is involved in high‐density lipoprotein (HDL) formation (Fitz et al., 2012). APP/PS1 mice with APOE4 insertion and ABCA1^−/+^ exhibited memory impairments and increased Aβ deposition (Fitz et al., 2012). Consistent with human studies, the CAPN10 gene plays an important role in DM in mice (Cheverud et al., 2010), and it may indirectly increase the risk of AD by contributing to T2DM.

### 1.7 Animal model treatment‐related insights

#### 1.7.1 Insulin

Insulin is a hormone produced primarily by pancreatic β cells (Rorsman & Ashcroft, 2018), although it has been shown that neurons can produce insulin (Blázquez et al., 2014; Gray et al., 2014). Insulin mRNA is found in brain regions relevant to AD such as the hippocampus (i.e., CA1 and CA3) (Devaskar et al., 1994). Elevated glucose levels initiate the synthesis and release of insulin via pancreatic β cells and promote cellular glucose uptake for energy generation. Downstream insulin signalling pathways include their role in protein transcription and synthesis, regulation of apoptosis and modulation of lipid synthesis in the brain (Arnold et al., 2018). Insulin plays a large role in brain development, neuronal health, and brain ageing. Both insulin and insulin‐like growth factor (IFG‐1) can modulate the proliferation, differentiation and survival of neural stem cells (Spinelli et al., 2019).

IRs are highly expressed on various cell types in the brain (i.e., neurons, microglia, oligodendrocytes, etc.) and across several brain regions including the hypothalamus, olfactory bulb, cerebellum, striatum, cortex and hippocampus (Arnold et al., 2018). IRs are highly localized in both pre‐ and post‐synaptic areas, playing an important role in neuroplasticity (Abbott et al., 1999; Bockmann et al., 2002; Mielke et al., 2006; Werther et al., 1989).

Insulin in the brain can promote neurite growth, regulates the expression and localization of γ‐aminobutyric acid (GABA), *N*‐methyl‐*d*‐aspartate (NMDA) and α‐amino‐3‐hydroxy‐5‐methyl‐4‐isoxazolepropionic acid (AMPA) receptors, and is involved in synaptic plasticity (i.e., LTP and LTD) in the hippocampus (van der Heide et al., 2005). Insulin helps maintain and promote excitatory synapses (Chiu et al., 2008) and dendritic spines (Lee, Huang, & Hsu, 2011) and can promote neuronal health and survival by inhibiting apoptosis (Arnold et al., 2018; Kim & Han, 2005). Neurons do not depend on insulin‐dependent GLUT‐4 receptors for glucose uptake as required for glucose uptake in peripheral cells. Instead, insulin‐independent receptors (e.g., GLUT‐3) are expressed on neurons and—indirectly activated via NMDA receptors—facilitate neuronal glucose uptake (Talbot et al., 2012b; Uemura & Greenlee, 2006).

Insulin and IRs decrease with normal ageing and in AD. The binding of insulin to IRs is directly associated with the production of insulin‐degrading enzyme (IDE). IDE is a key enzyme involved in the degradation of Aβ (Farris et al., 2003). IR activation suppresses the activation of GSK3β, a kinase involved in the phosphorylation of tau (Murtishaw et al., 2018). These mechanisms may explain why insulin treatment rescued learning and memory deficits in rodents (Adzovic et al., 2015; Steinmetz et al., 2016). Six weeks of treatment with intranasal insulin rescued brain insulin signalling dysfunction, ameliorated cognitive impairments, inhibited JNK activation, increased neurogenesis and reduced Aβ accumulation and plaques in 4.5‐month‐old APP/PS1 mice (Mao et al., 2016). Additional data, from an amyloid and tau mouse model of AD (3x‐Tg‐fAD mice) treated with intranasal insulin for 2 months, demonstrated improved memory (in novel object recognition task and MWM), reduced depression‐like behaviour (via tail suspension and forced swim tests) and decreased hyperphosphorylated tau, Aβ oligomers and 3‐nitrotyrosine in the frontal cortex and hippocampus (Barone et al., 2019). Additional data showed that ICV administration of insulin significantly decreased inflammatory markers in the hippocampus and improved spatial memory performance (Adzovic et al., 2015). It remains to be determined if the benefits of insulin administration are impacted when IR insensitivity is present in AD, aging or T2DM.

Soluble Aβ oligomers can impair insulin signalling via downregulation of IRs on neurons (Zhao et al., 2008). Elevated brain insulin initiates increased levels of IDE. Studies with mouse models of both T2DM and AD demonstrate that activating peroxisome proliferator‐activated receptor γ (PPARγ) and the adenosine monophosphate (AMP)‐activated protein kinase (AMPK) pathways significantly increase IDE leading to decreased Aβ levels and rescue of recognition and learning memory deficits (Li et al., 2018).

#### 1.7.2 Metformin

Metformin is the most utilized medication to treat T2DM as it decreases hepatic glucose production, decreases intestinal glucose absorption and increases insulin sensitivity. The effects of metformin in AD animal models, however, have not been entirely consistent across experiments. Studies suggest that metformin may have beneficial effects in age‐related diseases including AD (Rotermund et al., 2018). For example, mice with STZ‐induced hyperglycaemia given metformin showed reduce levels of phosphorylated tau and Aβ plaque burden in the hippocampus, decreased phosphorylated GSK‐3β in the cortex and improved learning and memory (Oliveira et al., 2021). In another study, administration of metformin led to a reduction of Aβ levels, improved learning and memory (i.e., MWM and Y‐Maze), enhanced mRNA expression of genes involved in synaptic plasticity (i.e., brain‐derived neurotrophic factor [BDNF]), deceased oxidative stress (i.e., malondialdehyde and superoxide dismutase), reduced inflammation (i.e. IL‐1β and IL‐6) and increased IDE protein levels in APP/PS1 mice (Lu et al., 2020). Long‐term metformin treatment in HFD aged C57BL/6J mice found that treatment with metformin prevented spatial learning and memory deficits (Allard et al., 2016). HFD mice given metformin showed enhanced glucose tolerance, as well as decreased oxidative stress and inflammation (Lennox et al., 2014). Administration of metformin to HFD rats led to reduced peripheral insulin resistance, decreased brain and plasma markers of oxidative stress, improved mitochondrial function and prevention of learning and memory impairments (Pintana et al., 2012). Senescence‐accelerated mouse‐prone 8 (SAMP8)—an AD mouse model—injected with metformin for 8 weeks exhibited improved learning and memory and decreased accumulation of APPc99 and hyperphosphorylated tau (Farr et al., 2019). Chronic metformin treatment rescued spine density, LTP and spatial memory in APP/PS1 mice via the suppression of cdk5 (Wang et al., 2020). Metformin also improves cognition of aged mice by promoting cerebrovascular integrity, enhanced glycolysis in blood and neurogenesis (Zhu et al., 2020).

However, not all studies have found beneficial responses to metformin in animal models of AD. Db/db mice injected with metformin did not exhibit improved spatial learning and memory (Li et al., 2012). One study found that metformin treatment in P301S mice—a tauopathy mouse model—showed reduced hyperphosphorylated tau but simultaneously exhibited increased tau aggregation (Barini et al., 2016). Similarly, short‐term metformin treatment reduced hyperphosphorylated tau but simultaneously promoted expression and processing of APP and BACE‐1 (Kickstein et al., 2010; Picone et al., 2015). The inconsistency observed in studies of the effects of metformin on AD‐related changes may be due to differing diet compositions as well as mechanistic differences in the various animal models.

#### 1.7.3 PPAR‐gamma agonists

Peroxisome proliferation‐activated receptors (PPARs) have a role in cellular functions relevant to T2DM and AD. PPARs are a type of ligand‐inducible nuclear hormone receptor superfamily that are regulated by steroids and lipid metabolites (Heneka et al., 2011; Nicolakakis & Hamel, 2010). PPARs are involved in lipid storage, adipocyte differentiation and glucose homeostasis in all organs, including the brain (Heneka et al., 2011; Nicolakakis & Hamel, 2010). Three isoforms of PPRAs exist (α, γ and β/δ), each encoded by different genes (Nicolakakis & Hamel, 2010). PPARγ has a role in insulin sensitizing effects of the PPARγ agonists thiazolidinediones, a class of oral anti‐diabetic agents (Lehmann et al., 1995; Nicolakakis & Hamel, 2010). PPARγ has relevance to both T2DM and AD, as PPARγ can regulate obesity, diabetes and neuroinflammation (de Carvalho et al., 2021). In animal models, PPARγ agonists have been observed to reduce microglial and astrocytic activation in the hippocampus and cortex, decrease BACE‐1 mRNA and protein levels and reduce Aβ deposits in the hippocampus and cortex (Heneka et al., 2005). PPARγ plays an important role in mitochondrial function and biogenesis, fatty acid storage, energy metabolism and antioxidant defence (Rodríguez‐Pascau et al., 2021). More recently, bis(ethylmaltolato)‐oxidovanadium (IV) (BEOV), a vanadium compound, was shown to be involved in DM and AD via PPARγ activity (He et al., 2021). Administration of BEOV in APP/PS1 mice significantly decreased levels of TNF‐α, IL‐6, IL‐1β, NO synthase and cyclooxygenase‐2 in hippocampus of APP/PS1 mice (He et al., 2021). Furthermore, these effects were observed in BV2 microglia cell cultures with Aβ (He et al., 2021). BEOV reduced Aβ levels and improved learning and memory in APP/PS1 mice (He et al., 2021).

There are several endogenous and synthetic agonists with a spectrum of affinity for PPARγ. Natural PPARγ agonists with high affinity include linoleic acid (9‐ and 13‐HODE), prostaglandin 15‐Deoxi‐Delta(12,14)‐prostaglandin J(2) (15d‐PGJ(2)) (Cocca et al., 2009; Khan et al., 2019), as well as gamolenic acid, eicosapentaenic acid, polyunsaturated fatty acid metabolites and others (Khan et al., 2019). Synthetic PPARγ agonists primarily include thiazolidinediones (e.g., pioglitazone [Actos]: troglitazone [Rezulin], ciglitazone and rosiglitazone [Avandia]) (Khan et al., 2019), as well as ibuprofen, indomethacin, flurbiprofen and others (Khan et al., 2019). APP/PS1 mice administered pioglitazone showed reduced microglia and astrocyte activation and reduced Aβ plaques in the hippocampus (Mandrekar‐Colucci et al., 2012). Pioglitazone increased microglial Aβ phagocytosis in the hippocampus in an AD mouse model (Yamanaka et al., 2012). Pioglitazone was shown to increase levels of APOE‐related genes, decrease proinflammatory genes and decrease Aβ levels in the hippocampus of an AD mouse model (Skerrett et al., 2015). Pioglitazone reduced tau phosphorylation at multiple sites in the cortex and CA1 region of the hippocampus and improved cognition in 3xTg mice (Adler et al., 2014). Similarly, administration of rosiglitazone reduced astrocytic and microglial activation, Aβ oligomers and aggregates, and spatial memory impairments in APP/PS1 mice (Toledo & Inestrosa, 2010). More recently, telmisartan, an antagonist for angiotensin receptor II type 1, commonly used for hypertension treatment, was shown to activate PPARγ with anti‐inflammatory and anti‐apoptotic effects (Khan et al., 2019). Telmisartan improved memory deficits seen in AD mouse models generated by ICV injection of STZ (Singh et al., 2013) and ICV injection of Aβ (Khan et al., 2019; Shindo et al., 2012; Tsukuda et al., 2009).

#### 1.7.4 GLP‐1 agonists

Glucagon‐like peptide 1 (GLP‐1) is a hormone that is released by the gut, is involved in the gut/brain axis, protects insulin‐producing pancreatic β cells and assists in insulin secretion (Cabou & Burcelin, 2011). GLP‐1 can directly modulate neurotransmitter release, is involved in LTP and protects synapses involved in LTP from Aβ oligomer‐induced damage (Gault & Hölscher, 2008). GLP‐1 reduces oxidative stress, is involved in autophagy regulation and exhibits anti‐inflammatory protective functions (e.g., anti‐inflammatory signalling) in the CNS (Li et al., 2009). GLP‐1 signal transduction is mediated by the GLP‐1 receptor (GLP‐1R), a G‐protein‐coupled receptor (Grieco et al., 2019). GLP‐1R can activate the phosphatidylinositol 3‐kinase/protein kinase B (PI3K/AKT) pathway leading to protection against apoptosis and inhibition of pro‐inflammatory cytokines (Farilla et al., 2003; Grieco et al., 2019; Tramutola et al., 2017; Yang et al., 2018).

GLP‐1R agonist agents (e.g., exenatide, lixisenatide, liraglutide, semaglutide, etc.) lower glucose levels and reduce cognitive deficits observed in T2DM (Aroda, 2018; Gomez‐Peralta & Abreu, 2019; Grieco et al., 2019). Administration of exenatide to rats reduced neuroinflammation (i.e., TNF‐α) (Solmaz et al., 2015) and rescued LTP from Aβ‐induced compromise of hippocampus function (Wang et al., 2016, p. 4).

Administration of lixisenatide reduced NFTs, Aβ plaques and chronic neuroinflammation in the hippocampus of APP/PS1/tau female mice (Cai et al., 2018). The effects of lixisenatide are via the PI3K/AKT/GSK‐3β signalling pathway and can prevent spatial memory and synaptic insults that are induced by Aβ oligomers (Cai et al., 2014). Peripheral administration of lixisenatide in mice on HFDs showed improvement of recognition memory, increased numbers of immature neurons in the dentate gyrus and upregulation of hippocampal expression of neurotrophic tyrosine kinase receptor type 2 (NTRK2) and mammalian target of rapamycin (mTOR) involved in modulating synaptic plasticity and LTP (Lennox et al., 2014).

Administration of liraglutide to mice with STZ‐induced hyperglycaemia resulted in improved learning and memory and reduced neuronal death in the hippocampus (Palleria et al., 2017). Treatment with liraglutide before administration of STZ led to neuroprotective effects against STZ‐related hippocampal neuronal death and cognitive impairments associated with the AMPK/mTOR signalling pathway (Kong et al., 2018). Liraglutide prevented tau hyperphosphorylation in the hippocampus of *db/db* mice via activating insulin signalling pathways and suppressed GSK‐3β activation (Ma et al., 2015). Liraglutide has been reported to reduce tau hyperphosphorylation, prevent learning and memory impairments, and alleviate the structural changes of pyramidal neurons in the hippocampus of mice with ICV injection of Aβ (Qi et al., 2016)

A novel dual GLP‐1 and glucose‐dependent insulinotropic polypeptide (GIP) combination (i.e., DA‐JC4) prevented hyperphosphorylation of tau, reduced chronic neuroinflammation, decreased apoptotic signalling and improved IR sensitivity in ICV‐injected STZ rats (Shi et al., 2017). A GLP‐1/gastric inhibitory polypeptide dual agonist (DA5‐CH) reduced Aβ plaque load and phosphorylated tau protein and improved learning and memory in APP/PS1 mice (Grieco et al., 2019). A triple receptor agonist that activated GIP‐1, GIP and glucagon receptors simultaneously significantly reduced Aβ accumulation, neuroinflammation (e.g., activated astrocytes and microglia) and mitochondrial oxidative stress in the hippocampus and cortex of APP/PS1 mice; the animals exhibited improved learning and memory (Tai et al., 2018).

#### 1.7.5 Commentary on non‐clinical and animal model observations linking T2DM and AD

**FIGURE 1:**
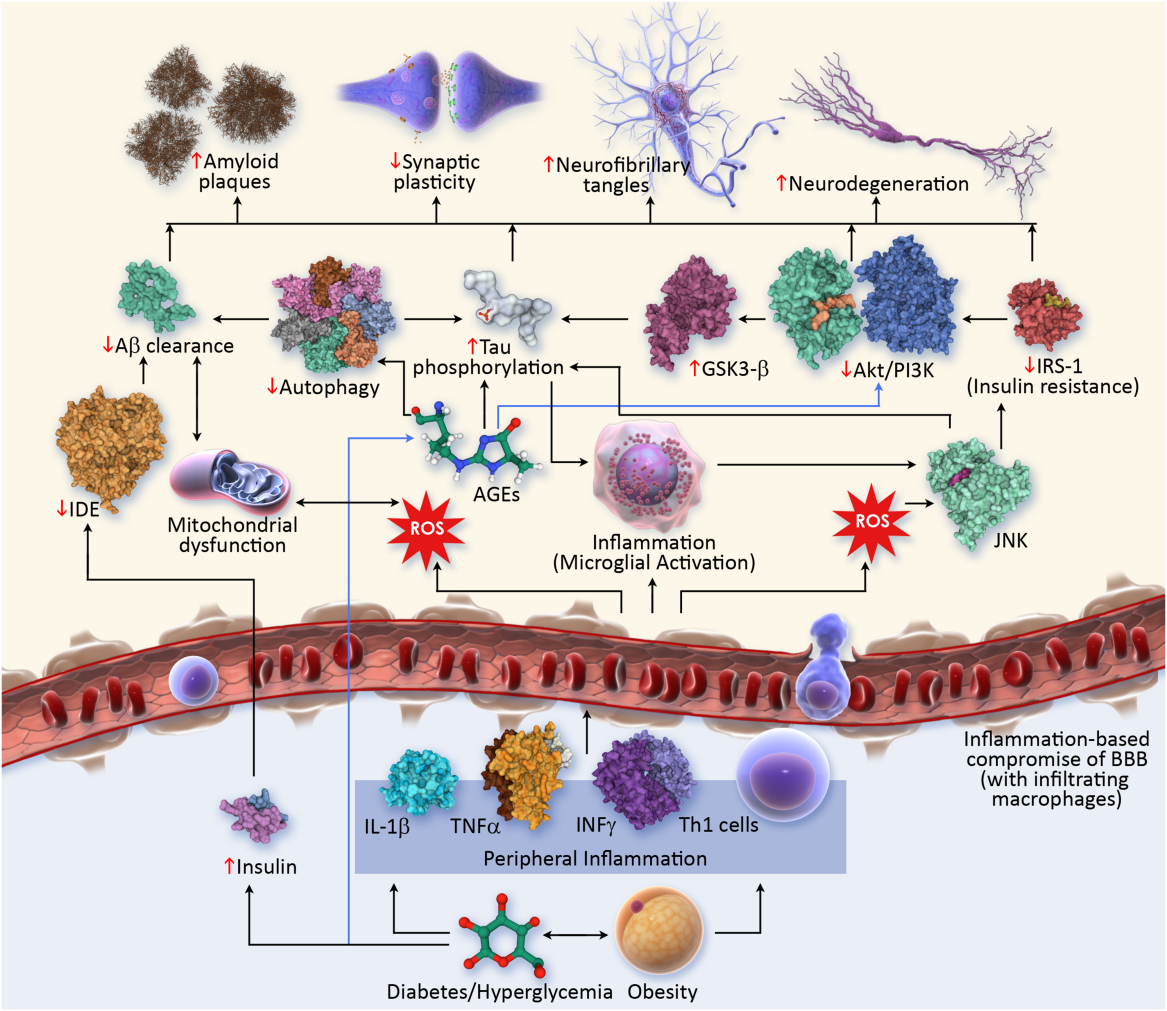
Pathophysiological links between T2DM and AD (AD, Alzheimer's disease; AGEs, advanced glycation end products; Akt, protein kinase B; BBB, blood–brain barrier; IDE, insulin degrading enzyme; IL1‐B, interleukin 1 B; INF, interferon‐gamma; IRS1, insulin receptor substrate 1; GSK3B, glycogen synthase kinase‐3B; JNK, c‐Jun N‐terminal kinase; PI3K, phosphoinositide 3 kinase; ROS, reactive oxygen species; T2DM, type 2 diabetes mellitus; Th1, T helper cells 1; TNF‐α, tumour necrosis factor‐α)

The non‐clinical studies reviewed provide an overview of the complex relationship between AD and T2DM. There is shared biology at the level of insulin resistance, IDE function, vascular effects, inflammation and genetic influences. Animal models have been informative regarding the adverse influences of T2DM features (e.g., hyperglycaemia and insulin resistance) on AD pathology, either by inducing AD‐like changes or exacerbating AD pathology in AD models. Animal studies provide substantial insight into the mechanistic effects of anti‐diabetes medications and how they affect both T2DM and AD‐related pathology and behaviour. Figure 1 shows the complex interaction of T2DM and AD biological mechanisms.

### 1.8 Clinical trials of anti‐diabetic drugs for the treatment of AD

The many links between diabetes and AD suggest that therapies for T2DM might be beneficial in the treatment of AD. We performed a review of clinicaltrials.gov, a comprehensive registry of clinical trials identifying all T2DM therapies in trials for AD. We identified 10 trials assessing the impact of insulin in AD and 20 AD trials involving 10 non‐insulin anti‐diabetic agents conducted since 2006. Among the insulin trials: eight were completed, one was terminated, and one was withdrawn. Among the non‐insulin anti‐diabetic agents, seven trials are on‐going, eight trials have been completed, and five trials were terminated. Table 1 provides the details of the insulin trials including the trial phase, intended number of participants, Mini‐Mental Status (MMSE) score range of the eligible participants, treatment duration in the trial and clinical and biomarker outcomes included in the trial. Table 2 provides the same information for the non‐insulin anti‐diabetic agents. Classes of agents represented in the trials include insulin, biguanides (e.g., metformin and metformin‐extended release [XR]), thiazolidinediones (e.g., pioglitazone and rosiglitazone), sodium‐glucose co‐transporter 2 (SGLT2) inhibitors (e.g., dapagliflozin and empagliflozin) and GLP‐1 analogs (e.g., exenatide, liraglutide and semaglutide).

**TABLE 1:** Characteristics of trials of insulin for the treatment of Alzheimer's disease

Trial number	Agent	Date registered	Trial status	Trial phase	Enrolment	MMSE range	Treatment duration	Primary clinical outcomes	Outcome biomarkers
NCT00581867	Insulin aspart	12/19/07	Completed	Phase 1 Phase 2	31		Single dose	fMRI measure of hippocampal activation	
NCT02503501	Insulin glulisine	7/14/15	Terminated	Phase 2	49	MoCA 18–27	26 weeks	ADAS‐cog13 CDR	CSF Aβ 42 CSF tau CSF phosphotau cerebral glucose metabolism via FDG PET
NCT01145482	Insulin aspart	6/15/10	Completed	Not applicable	12	>15	2 doses	Cerebral glutamate concentration	
NCT02462161	Insulin aspart	6/1/15	Completed	Phase 1	24		12 weeks	ADAS‐cog MCI scores	CSF Aβ CSF tau Plasma Aβ Plasma tau Cortical thickness in AD‐vulnerable regions Inflammatory markers
NCT01636596	Insulin lispro	7/6/12	Withdrawn	Not applicable	0	≥15	26 weeks	MMSE CDT	Cerebral glucose metabolism via FDG PET Basal metabolism
NCT01436045	Insulin glulisine	9/16/11	Completed	Phase 2	12	18–26	Single dose	Trails B ‐ seconds Trails B ‐ errors Cognitive performance via RBANS	
NCT01767909	Insulin regular (Humulin R)	1/10/13	Completed	Phase 2 Phase 3	240	≥20	26 weeks	ADAS‐cog12	CSF Aβ CSF tau Degree of hippocampal/entorhinal atrophy
NCT01595646	Insulin detemir	5/8/12	Completed	Phase 2	37		16 weeks	Verbal memory composite (delayed story recall and Buschke selective reminding test)	CSF Aβ CSF tau CSF TTau‐P181/Aβ 42 ratio Plasma Aβ Plasma tau Cerebral blood flow via MRI OGTT
NCT01547169	Insulin detemir	2/7/12	Completed	Phase 2	60		3 weeks	Verbal memory composite (immediate + delayed story recall and immediate + delayed list recall)	Plasma Aβ Plasma tau OGTT
NCT00438568	Insulin regular (Novolin R)	7/21/2007	Completed	Phase 2	173		16 weeks	Changes in cognition	CSF Aβ Plasma Aβ Cerebral glucose metabolism via PET

Abbreviations: Aβ, amyloid beta; ADAS‐cog, Alzheimer's Disease Assessment Scale ‐ Cognitive Subscale; CSF, cerebrospinal fluid; CT, computed tomography; FDG PET, fluorodeoxyglucose positron emission tomography; GDS, Global Deterioration Scale; MCI, mild cognitive impairment; MMSE, Mini‐Mental State Exam; MRI, magnetic resonance imaging; MoCA, Montreal Cognitive Assessment; OGTT, Oral Glucose Tolerance Test; RBANS, Repeatable Battery for the Assessment of Neuropsychological Status.

**TABLE 2:** Characteristics of trials of non‐insulin anti‐diabetic agents tested for the treatment of Alzheimer's disease

Trial number	Agent	Date registered	Trial status	Trial phase	Enrolment	MMSE range	Treatment duration	Primary clinical outcomes	Outcome biomarkers
NCT04098666	Metformin XR	9/19/19	Recruiting	Phase 2 Phase 3	370	≥20	104 weeks	FCSRT	Brain Aβ SUVR, brain tau SUVR, plasma Aβ
NCT01965756	Metformin	10/16/13	Completed	Phase 2	20	>21	16 weeks	ADAS‐cog	CSF Aβ, Total tau, phosphorylated tau concentration
NCT00620191	Metformin	2/7/08	Completed	Phase 2	80	≥20	52 weeks	ADAS‐cog, Total recall score in SRT	Plasma Aβ‐42
NCT00982202	Pioglitazone	9/22/09	Completed	Phase 2	25	12–26	78 weeks	ADAS‐cog, CDR‐SB, CIBIC‐plus	Laboratory abnormalities
NCT01931566	Pioglitazone	8/26/13	Terminated	Phase 3	3,494	≥25	Up to 260 weeks	Time to diagnosis of MCI‐AD	
NCT02284906	Pioglitazone	11/4/14	Terminated	Phase 3	40	≥25	104 weeks	Change from extension study baseline in composite score of cognitive test battery	
NCT04251182	T3D‐959	1/24/20	Recruiting	Phase 2	256	14–26	24 weeks	ADAS‐cog11, ADCS‐CGIC	Plasma Aβ 42/40 ratio
NCT00428090	Rosiglitazone XR	1/25/07	Completed	Phase 3	862	10–23	24 weeks	ADAS‐cog, CIBIC+ global functioning Total score	HgbA1c, body weight
NCT00348309	Rosiglitazone XR	6/30/06	Completed	Phase 3	1,496	10–26	52 weeks	ADAS‐cog, CDR‐SB	HgbA1c
NCT00490568	Rosiglitazone XR	6/21/07	Terminated	Phase 2	1,461	10–26	76 weeks	AEs	HgbA1c, body weight
NCT00348140	Rosiglitazone XR	6/30/06	Completed	Phase 3	1,468	10–26	54 weeks	ADAS‐cog, CDR‐SB	HgbA1c, body weight
NCT00550420	Rosiglitazone XR	10/25/07	Terminated	Phase 3	331	10–26	52 weeks	AEs	HgbA1c, body weight
NCT01374438	MSDC‐0160 (Mitoglitazone)	6/14/11	Completed	Phase 2	29	≥20	12 weeks	Cerebral glucose metabolic rate	High molecular weight adiponectin
NCT03801642	Dapagliflozin	12/21/18	Recruiting	Phase 1 Phase 2	48	15–26	12 weeks	Cerebral *N*‐acetylaspartate	Cerebral glucose metabolism via FDG PET, HgbA1c, Plasm β‐hydroxybutyrate, activated AKT levels, mTOR phosphorylation, platelet cytochrome oxidase activity, MCP‐1, Eotaxin‐1, TNF‐α, CRP
NCT03852901	Empagliflozin	2/22/19	Recruiting	Phase 1	100		2 weeks	Ketone bodies	Ketone bodies
NCT01255163	Exenatide	12/4/10	Terminated	Phase 2	57	>20	78 weeks	Incidence of nausea	CSF total tau, CSF p181‐tau, CSF Aβ‐42, BMI
NCT01843075	Liraglutide	4/24/13	Active, not recruiting	Phase 2	204	≥20	52 weeks	Cerebral glucose metabolic rate	Cortical amyloid, tau deposition, microglial activation
NCT01469351	Liraglutide	11/1/11	Completed	Not applicable	34	≥18	26 weeks	Intracerebral amyloid deposition via PIB PET scan	Intracerebral amyloid deposition, glucose uptake in CNS by FDG PET
NCT04777396	Semaglutide	2/26/21	Recruiting	Phase 3	1,840	≥22	173 weeks	CDR‐SB	High sensitivity CRP level
NCT04777409	Semaglutide	2/26/21	Recruiting	Phase 3	1,840	≥22	173 weeks	CDR‐SB	

Abbreviations: Aβ, amyloid beta; AD, Alzheimer's disease; ADAS‐cog, Alzheimer's Disease Assessment Scale ‐ Cognitive Subscale; ADCS, Alzheimer's Disease Cooperative Study; ADFACS, Alzheimer's Disease Functional Assessment of Change Scale; ADL, Activities of Daily Living; ADLMCI, Activities of Daily Living Scale for Mild Cognitive Impairment; AEs, adverse events; BMI, body mass index; CDR‐SB, Clinical Dementia Rating Scale Sum of Boxes; CGIC, Clinical Global Impressions of Change; CIBIC‐Plus, Clinician's Interview‐Based Impression of Change Plus Caregiver Input; CRP, C‐reactive protein; CSF, cerebrospinal fluid; FCSRT, Free and Cued Selective Reminding Test; FDG PET, fluorodeoxyglucose positron emission tomography; HgbA1c, haemoglobin‐A1c; MCI, mild cognitive impairment; MCP‐1, monocyte chemotactic protein 1; MMSE, Mini‐Mental State Exam; mTOR, mammalian target of rapamycin; SRT, spaced retrieval training; SUVR, standardized uptake value ratio; TNF, tumour necrosis factor; XR, extended release.

#### 1.8.1 Insulin

In most insulin trials investigating effects on memory or AD, insulin is delivered by intranasal administration to avoid hypoglycemia (Craft et al., 2012a). A trial of memory effects 15 minutes after intranasal insulin in normal controls or patients with mild‐moderate AD dementia or MCI (*N* = 61) showed improvement in those without the APOE‐4 genotype (Reger et al., 2006). A 21‐day randomized controlled study (*N* = 25) using intranasal administration of insulin found improved memory, attention and function (Reger et al., 2008). In a study comparing two doses of insulin with placebo, Craft and colleagues showed an improvement in memory in the low‐dose group (Craft et al., 2012a). There were no effects of insulin on CSF amyloid levels and modest effects on stabilizing decline of hypometabolism on fluorodeoxyglucose (FDG) and positron emission tomography (PET) among participants treated for 4 months (*N* = 104) (Craft et al., 2012a). A 4‐month study (*N* = 36) comparing regular insulin, long‐acting insulin, and placebo found improved memory and change in the Aβ42/p‐tau ratio in the CSF in those receiving regular insulin (Craft et al., 2017). In a larger 12‐month study (*N* = 289), no drug‐placebo differences were observed on the AD Assessment Scale – cognitive subscale (ADAS‐cog) or CSF markers of AD (Craft et al., 2020). Treatment of participants with mild to moderate AD given intranasal insulin for 2 days following a course of high‐dose Vitamin D demonstrated improved memory on some assessments and not others (Stein et al., 2011). A blinded cross‐over study of rapid‐acting insulin (i.e., glulisin insulin) in patients with the APOE4 genotype reported no benefit for memory function (Rosenbloom et al., 2014).

No insulin trials have been rigorously conducted using contemporary standards of diagnosis (e.g., biological confirmation of the diagnosis of AD). Many of the trials have involved relatively small numbers of patients. Some of the trials have shown measurable effects on recent memory; few have shown effects on other cognitive functions, global ratings or functional assessments. Overall, trials of insulin for the treatment of AD do not comprise a body of evidence in favour of this intervention in patients with symptomatic AD.

#### 1.8.2 Biguanides (metformin, metformin‐XR)

Metformin was assessed for its cognitive benefits in a population of patients with MCI and obesity. Eighty participants were randomized to metformin or placebo. Few subjects (10%) tolerated the intended dose of 1,000 mg twice daily. Primary outcomes were the Selective Reminding Test (SRT) and the ADAS‐cog. At trial end, there was a significant drug‐placebo difference in favour of metformin on the SRT; other measures including FDG PET showed not drug‐placebo difference (Luchsinger et al., 2004). In another trial, 20 non‐diabetic subjects with MCI or mild AD dementia were randomized to active treatment or placebo in an 8‐week cross‐over study. The metformin group exhibited significantly improved executive function and a trend towards improved memory and learning; no drug‐placebo difference was seen on other cognitive assessments or on measures of cerebral blood flow (Koenig et al., 2017). Given its pleiotropic effects, metformin has been proposed as an anti‐aging and multi‐organ disease modifying intervention; trials have been designed in which cognition would be included among several age‐related outcomes (Justice et al., 2018).

Metformin has not been extensively assessed in clinical trials. Trials for which results are available offer modest support for further testing in AD. Improved diagnostic specificity, larger sample sizes, and longer durations of treatment are required to thoroughly test metformin for possible efficacy in AD.

#### 1.8.3 Thiazolidinediones (pioglitazone, rosiglitazone)

Thiazolidinedione PPARγ agonists exert anti‐diabetic effects, lowering peripheral insulin and increasing insulin sensitivity. In a randomized unblinded study of 32 participants with MCI or mild to moderate AD dementia treated for 6 months with pioglitazone, those on active treatment had significantly improved scores on the ADAS‐cog and a test of logical memory. No drug‐placebo difference was observed on the MMSE (Hanyu et al., 2009). In a 6‐month randomized unblinded study of 42 patients with mild AD dementia and T2DM, those on active treatment exhibited improved cognition and increased blood flow in the parietal lobes not seen in those assigned to placebo. A decrease in fasting peripheral insulin levels suggested improved insulin sensitivity in those on pioglitazone (Sato et al., 2011). A randomized, double‐blind 18‐month trial of 25 non‐diabetic participants with AD dementia showed pioglitazone to be safe. No cognitive benefits were observed (Geldmacher et al., 2011).

Pioglitazone has been assessed in a Phase 3 AD prevention trial (the TOMMORROW trial) intended to determine whether treatment would delay time to progression to MCI due to AD. Eligible participants were stratified based a combination of *TOMM40* rs 10524523 genotype, APOE genotype and age, with high‐risk individuals receiving low‐dose pioglitazone or placebo and low‐risk individuals receiving placebo. A sample size of 2,346 participants was estimated to be required to assess the intended outcome (Burns et al., 2019). During the study, the sample size was reduced and the trial shortened. No drug‐placebo difference was observed in time to progression to MCI due to AD at the time of trial termination.

Rosiglitazone is a PPARγ agonist assessed for possible efficacy in the treatment of AD. A 6‐month Phase 2 proof‐of‐concept study with 30 participants randomized to rosiglitazone or placebo demonstrated significant improvement in delayed recall and selective attention in the active treatment group (Watson et al., 2005). A 6‐month Phase 2 randomized controlled trial including 511 participants assessed cognitive and global outcomes for 2, 4 and 8 mg of rosiglitazone compared with placebo. No drug‐placebo difference was observed on the primary outcomes for any dose. An exploratory analysis of APOE4 non‐carriers showed significant benefit on the ADAS‐cog for those on active treatment (Risner et al., 2006). A follow‐up 24‐week Phase 3 study comparing rosiglitazone 2 mg, rosiglitazone XR 8 mg, donepezil and placebo (*N* = 693) found no drug‐placebo difference on cognitive or global measures in the total population or the APOE4 non‐carriers. Participants in the donepezil arm of the study had no drug‐placebo difference on the ADAS‐cog raising questions about the trial conduct. The participants receiving donepezil exhibited a significant benefit of treatment as measured on the Clinician's Interview‐Based Impression of Change with Caregiver Input (CIBIC+) (Gold et al., 2010). In a 12‐month randomized controlled trial of rosiglitazone XR compared with placebo using FDG PET as the primary outcome, participants on active treatment showed a non‐significant increase in glucose metabolism in the first month of treatment. No drug‐placebo differences were observed in decline in glucose metabolism, rate of cerebral atrophy, or cognitive measures (Tzimopoulou et al., 2010). Two Phase 3 48‐week randomized controlled trials including nearly 3,000 patients found no drug‐placebo difference on cognitive or global measures in the total study population, APOE4 non‐carriers or all patients except APOE4 homozygotes (Harrington et al., 2011). A follow‐up study of these trials showed that a predefined 6‐protein metabolic and inflammatory biomarker panel (i.e., IL‐6, IL‐10, C‐reactive protein [CRP], TNF‐α, heart‐type fatty acid‐binding protein 3 [FABP‐3] and pancreatic polypeptide [PPY]) correctly identified a treatment response among those receiving rosiglitazone with 98% accuracy (O'Bryant et al., 2021).

These large studies of rosiglitazone in mild‐moderate AD dementia demonstrate no treatment benefit. The post hoc analysis of responders with a characteristic biomarker profile leaves open the possibility that within the population, there is a subgroup of rosiglitazone‐responsive individuals.

#### 1.8.4 SGLT2 inhibitors (dapagliflozin, empagliflozin)

SGLT2 inhibitors reduce blood glucose levels by inhibiting glucose reabsorption by the kidney, inducing glucosuria. They reduce fasting and postprandial blood glucose levels, body weight and blood pressure. SGLT2 inhibitors reduce mTOR kinase activity that may contribute to lysosomal and mitochondrial dysfunction in AD. mTOR activity is associated with BBB endothelial cell dysfunction, tau hyperphosphorylation and Aβ plaque formation (Esterline et al., 2020). The first trials of SGLT2 inhibitors in AD have been inaugurated.

#### 1.8.5 Incretin mimetics/GLP‐1 analogs (exenatide, liraglutide, semaglutide)

The National Institute on Aging (NIA) with support from AstraZeneca, conducted an 8‐month, double‐blind, randomized, placebo‐controlled Phase 2 clinical trial to assess the safety and tolerability of exenatide in early AD. Eighteen participants with high probability of AD completed the entire study prior to its early termination. Exenatide was shown to be safe and well‐tolerated in this population. Among outcomes assessed, there were no differences compared with placebo for clinical and cognitive measures, magnetic resonance imaging (MRI) assessments of cortical thickness and volume, or biomarkers in CSF, or plasma. There was a reduction in Aβ42 in neuronal extracellular vesicles. The investigators note that the study was underpowered due to early termination, and firm conclusions regarding efficacy cannot be drawn (Mullins et al., 2019).

In a 26‐week, randomized, placebo‐controlled, double‐blind trial conducted in Demark and including 24 non‐diabetic participants with AD, those treated with liraglutide were noted to have no difference in amyloid deposition or cognition compared with those on placebo. The researchers note that in patients with long‐standing AD, the 26 weeks of liraglutide treatment had prevented the expected decline of regional cerebral glucose measure on FDG PET (Gejl et al., 2016).

Liraglutide's effect on cerebral glucose metabolism and cognitive function was assessed in a 12‐month, multicentre, randomized, double‐blind, placebo‐controlled, Phase 2 trial (Evaluating Liraglutide in AD [ELAD] study). Two hundred and four participants diagnosed with probable AD were enrolled (Femminella et al., 2019). No drug‐placebo differences were observed in the primary outcome of cerebral glucose metabolism or in the secondary outcomes of Clinical Dementia Rating ‐ Sum of Boxes (CDR‐SB) score and AD Cooperative Study – Activities of Daily Living (ADCS‐ADL) scale. An improvement was noted in the AD Assessment Scale – Executive version (ADAS‐Exec), and there was reduced loss of temporal lobe and total brain grey matter volume in participants receiving liraglutide (Alzforum, 2021).

Novo Nordisk A/S is currently recruiting for two trials investigating semaglutide in participants with early AD, the EVOKE and EVOKE+ trials. Participant diagnosis is confirmed by amyloid PET or CSF amyloid measures consistent with AD. The primary outcome is drug‐placebo difference in change in cognition from baseline using the CDR‐SB score (ClinicalTrials.gov, 2021). A secondary outcome is time to progression to AD dementia in those with MCI at trial baseline. Participants of these trials have amyloid PET at baseline to confirm the diagnosis of AD. EVOKE+ allows participants with subcortical cerebrovascular disease to enter the study, the EVOKE trial does not allow such participants.

#### 1.8.6 Trial commentary

Among the insulin trials, two were Phase 1 trials, seven were Phase 2 trials, and one was a Phase 3 trial (two trials did not have identified phases). The trials of non‐insulin anti‐diabetic agents included two Phase 1 trials, nine Phase 2 trials and nine Phase 3 trials (one trial had no declared phase). The non‐insulin agents in Phase 3 included metformin (one trial), pioglitazone (two trials), rosiglitazone (four trials) and semaglutide (two trials).

Some insulin trials showed clinical benefit in exploratory trials (Claxton et al., 2015; Craft et al., 2012b, 2017). Very acute trials with intravenous insulin suggested improvement on memory measures (Craft et al., 2003; Watson et al., 2009). These positive outcomes were not replicated in larger longer term studies (e.g., 6‐ to 12‐month duration). All the completed studies on the use of non‐insulin T2DM treatments for AD have been negative; seven of the 20 are currently ongoing. A trial with liraglutide showed a numerical but not significant stabilization of brain metabolism on FDGF PET contrasting with metabolic decline in the placebo group (Gejl et al., 2016). The observation supports the suggestion that GLP‐1 agonists have CNS effects despite not crossing the BBB.

Of the 20 non‐insulin trials of agents used to treat T2DM, only four confirmed the diagnosis of AD with amyloid biomarkers. Of the 10 insulin trials, none had confirmatory amyloid biomarkers assessed. Studies show that of patients recruited for trials of early AD (MCI due to AD and mild AD dementia) and exhibiting AD clinical features, 40% are excluded due to negative amyloid scans (Sevigny et al., 2016). Similarly, in a trial of mild‐moderate AD, 30% of patients with the mild AD phenotype and 15% of these diagnosed with moderate AD had negative amyloid imaging (Degenhardt et al., 2016). These observations suggest that most of the trials of insulin and of non‐insulin T2DM therapies not using diagnostic biomarkers likely have included 15%–40% of participants who lack the biology of AD; the canonical biology of AD—Aβ plaque formation—is absent in these amyloid negative participants. Non‐amyloid bearing participants do not have AD and exhibit slower decline, compromising the ability to observe treatment‐placebo differences (Ballard et al., 2019). Inclusion of non‐AD patients may have negatively affected the outcomes of these trials.

There has been only one AD prevention trial using an anti‐diabetic agent—an assessment of pioglitazone in cognitively normal individuals at increased genetic risk for AD (Burns et al., 2019). This study was terminated before the planned outcomes could be determined. Based on the increased risk for AD observed in epidemiologic studies of T2DM and the animal model studies demonstrating the ability to induce aspects of AD pathology with hyperglycaemia, additional long duration prevention trials are warranted.

Ten of the non‐insulin trials include individuals with MMSE scores of 20 or above consistent with early AD; the other trials include patients with mild‐moderate AD dementia. Entry criteria for the insulin trials often did not specify an MMSE range; of those providing this information, most of the trials included patients with mild or moderate AD dementia. No patients with severe dementia are included in any of the trials.

None of the insulin trials and two of the non‐insulin trials required participants to have risk factors for T2DM. In both trials including T2DM risks, AD with concomitant obesity was the identified trial population.

The primary outcomes of the trials commonly included the ADAS‐cog, CDR‐SB or measures of memory and delayed recall. Cerebral glucose metabolism measured by FDG PET, ketone bodies or adverse events were primary outcomes in some trials. Diabetes‐related biomarkers including haemoglobin‐A1c (HgbA1c) and CRP were included in eight of 20 non‐insulin trials and none of the insulin trials.

Trial duration and number of participants vary greatly among these studies. Insulin trials recruited between 12 and 240 participants and were from a few hours for single dose studies to 24 weeks in duration. Non‐insulin trials vary between short observation periods of 2 weeks (empagliflozin) to studies intended to have up to 5 years of exposure (pioglitazone). Non‐insulin trial populations have varied between 20 and 1,840 trial participants among completed or ongoing trials. The size and the duration of the trials are determined by the specific question being asked and the anticipated effect size of the therapy. Industry‐sponsored trials are typically larger and longer than academic or government‐sponsored trials. Short‐term trials can detect improvement above baseline and the possible cognitive enhancing properties of T2DM agents; they can assess short‐term effects on biomarkers that may predict benefits with longer term treatment. Trials of 12‐month duration or longer are required to collect clinical and biomarker data showing slowing of cognitive decline supportive of disease modification (Cummings, 2019; Cummings et al., 2018).

## 2 DISCUSSION

Many avenues of information link T2DM with the AD continuum. Animal models of T2DM with hyperglycaemia exhibit AD‐type pathological changes in the brain including tau hyperphosphorylation, tau aggregates and neuroinflammation (Engin & Engin, 2021; Murtishaw et al., 2018). Double and triple Tg mouse models of AD exhibit insulin resistance and energy dyshomeostasis similar to that observed T2DM patients (Velazquez et al., 2017). Amyloid pathology is more severe in several of the studies using AD model animals with concomitant physiological changes of T2DM. In humans, T2DM increases the risk for AD. T2DM is associated with obesity, and obesity is a risk for AD. Epidemiologic observations suggest that treatment of T2DM with some types of agents including rosiglitazone and GLP‐1 agonists is associated with a diminished risk for AD compared with those treated with other agents (Akimoto et al., 2020). The biology of AD includes insulin resistance similar to that seen in peripheral tissues of T2DM patients (Talbot et al., 2012b).

Peripheral inflammation stimulates neuroinflammation through transfer of inflammatory exosomes to the brain from the periphery and entry of peripheral inflammatory cells through a compromised BBB (Li et al., 2018; Pugazhenthi et al., 2017; Ransohoff, 2016; Zlokovic, 2008); both diabetes and obesity exhibit chronic low‐grade inflammation in association with insulin resistance (Osborn & Olefsky, 2012). Some classes of T2DM therapies—metformin and GLP‐1 agonists—reduce peripheral inflammation and this may contribute to both their anti‐diabetic properties and the reduction in AD incidence reported with their use (Lee & Jun, 2016; Saisho, 2015). GLP‐1 agonists may facilitate insulin entry in the brain, reduce insulin resistance, decrease neuroinflammation and restore neurogenesis in the absence of elevated peripheral inflammation suggesting that they may be useful in the treatment of non‐diabetic AD patients (Bae & Song, 2017; Cai et al., 2018).

Questions to be resolved in applying T2DM agents to AD are raised by non‐clinical and clinical observations. Animal models of the relationship of T2DM to AD most often involve STZ‐induced hyperglycaemia and investigation of the ensuing AD‐like pathological changes observed in the brain (Murtishaw et al., 2018). This is a strong model of the cognitive and pathological changes occurring in diabetes, diabetes as a risk factor for AD and brain changes in pre‐diabetes. It is less suited to the study of AD in the absence of T2DM. Most AD patients do not have T2DM, peripheral insulin resistance or hyperglycaemia, although an undetermined number may have pre‐diabetes. Among the studies reporting an increased incidence of AD in patients with T2DM, few had AD diagnostic confirmation with amyloid biomarkers, and—given the common occurrence of cerebrovascular disease in T2DM—the dementia syndromes observed may be AD, vascular dementia or mixed dementia (Ahtiluoto et al., 2010; R. Yang et al., 2019). The reductions in dementia incidence observed with treatment with T2DM agents typically occurred in samples observed for many years in registries or in long diabetes trials; this may translate into challenges showing reductions in dementia with shorter term trials and in patients without predisposing T2DM. Agents that reduce dementia incidence in T2DM cohorts do not necessarily exert effects on AD biology once the disease is expressed in the brain or may have limited effects that are difficult to demonstrate in trials. Some agents used for the treatment of T2DM are of high molecular weight and may pass the BBB in only limited amounts. More investigation of the central versus peripheral effects of these agents is needed to optimize effects on AD. Novel GLP‐1/GIP mimetics have shown neuroprotective effects in animal models of neurodegenerative disease and may warrant testing in next generation clinical trials (Holscher, 2018; Zhang et al., 2020).

An unresolved issue central to clinical trial design is whether to anticipate cognitive improvement as observed in short‐term (e.g., 6 months) cognitive enhancer‐type trials or to conduct disease‐modifying‐type trials that are longer and designed to determine if there is slowing of clinical decline (e.g., 12–24 months). Biomarkers relevant to cognitive enhancer‐type trials might include electroencephalography, FDG PET, of functional MRI. Biomarkers relevant to trials designed to show disease modification are those reflective of AD biology including measures of amyloid, tau, inflammation, and neurodegeneration. Anti‐diabetic agents may improve neuronal function producing cognitive enhancement; they may enhance neuronal survival leading to slowing of disease progression; or they may do both. Defining the expected response to these treatments is critical to designing trials most likely to capture a treatment effect. Trials in preclinical populations at‐risk for AD with DM or pre‐diabetes are another avenue of study of these agents.

New data on the relationship of T2DM and AD are accruing rapidly, and more insight into unresolved issues is anticipated. The identification of discrete mechanisms/signalling cascades that link T2DM and AD is needed to differentiate interventions for diabetic patients at risk for developing AD versus treatments targeting the core AD biology that overlaps with T2DM. If therapeutic benefits of agents used to treat T2DM can be extended to AD based on the many links between these two disorders, development of novel therapeutic regimens for patients with AD will be facilitated.

## CONFLICT OF INTEREST

JC has provided consultation to AB Science, Acadia, Alkahest, AlphaCognition, ALZPath, Annovis, AriBio, Artery, Avanir, Biogen, Cassava, Cerevel, Clinilabs, Cortexyme, Diadem, EIP Pharma, Eisai, GatehouseBio, GemVax, Genentech, Green Valley, Grifols, Janssen, Karuna, Lexeo, Lilly, Lundbeck, LSP, Merck, NervGen, Novo Nordisk, Oligomerix, Otsuka, PharmacotrophiX, PRODEO, Prothena, ReMYND, Renew, Resverlogix, Roche, Signant Health, Suven, Unlearn AI, Vaxxinity, VigilNeuro, Zai Laboratories pharmaceutical, assessment and investment companies. AO, JC and JK have no conflicts of interest.

## AUTHOR CONTRIBUTIONS

C.D. and D.E. verified the analytical methods. B.C. encouraged A.B. to investigate (a specific aspect) and supervised the findings of this work. All authors discussed the results and contributed to the final manuscript.

### PEER REVIEW

The peer review history for this article is available at https://publons.com/publon/10.1111/ejn.15619.

## Data Availability

All new data in this article are derived from clinicaltrials.gov and are freely publicly available. Our analyses as applied to the data are described in the methods section and can be reproduced. A web‐portal to enhance access to clinical trial data is under construction and will be available by contacting the lead author or by searching Alzheimer's Clinical Trial Innovation (ACTION) Initiative.
